# Implementation and Evaluation of a Social Networking Service–Based Mobile Patient-Generated Health Data System With Direct Electronic Medical Record Integration: Prospective Observational Study

**DOI:** 10.2196/81317

**Published:** 2026-06-23

**Authors:** Eunjoung Choi, Sunki Lee, Jinsung Jeon, Eung Ju Kim

**Affiliations:** 1 Cardiovascular center, Korea University Guro Hospital, 148 Gurodong-ro, Guro-gu, Seoul, 08308, Republic of Korea, 82 02-2626-3022, 82 02-2626-2024; 2 Department of Cardiology, College of Medicine, Korea University, Seoul, Republic of Korea; 3 Department of Cardiology, Asan Medical Center, Seoul, Republic of Korea

**Keywords:** patient-generated health data, electronic medical records, mobile health, heart failure, outpatient, patient satisfaction, patient engagement, documentation

## Abstract

**Background:**

Patient-generated health data (PGHD) can enhance patient-centered care by improving disease awareness and preparedness for clinical encounters. However, automated incorporation of PGHD into electronic medical records (EMRs), which is a prerequisite for broader clinical implementation, remains technically and administratively challenging.

**Objective:**

This study describes the development of *Miri-Alimi*, a PGHD collection platform that delivers mobile social networking service–based previsit questionnaires with automated transfer of structured patient responses into the EMR, and evaluates patient participation, EMR documentation quality, and user satisfaction in a cardiology outpatient clinic.

**Methods:**

This single-center observational study was conducted between August and November 2024 and included 751 consecutive cardiology outpatients, comprising 282 first-visit patients and 469 patients attending follow-up visits for heart failure. All eligible patients received a previsit electronic questionnaire link via KakaoTalk or multimedia messaging service prior to their scheduled visit. The primary outcomes were the overall survey response rate among all enrolled patients and EMR documentation completeness among follow-up patients with heart failure. Documentation quality was evaluated based on 3 prespecified parameters relevant to routine heart failure care—dyspnea, peripheral edema, and medication adherence status—and was quantified using an EMR completeness score ranging from 0 to 3. Secondary outcomes included patient and provider satisfaction assessed using postvisit 5-point Likert-scale surveys. Firth penalized logistic regression was used to evaluate the association between survey response status and EMR completeness, with adjustment for age and sex.

**Results:**

The response rate was 38.5% (289/751), including 48.9% (138/282) of new patients and 32.2% (151/469) of follow-up patients with heart failure. Responders were younger than nonresponders (mean 62.0, SD 15.7 years vs mean 69.8, SD 12.5 years; *P*<.001). Among the follow-up patients with heart failure, EMR completeness was higher among responders (median score 3, IQR 3‐3) than among nonresponders (median score 0, IQR 0‐1; *P*<.001). Patient satisfaction was high: 82.9% (63/76) to 92.1% (70/76) agreed that the system was appropriate, easy to use, and helpful, and 78.9% (60/76) completed the survey in <10 minutes. Both cardiologists and 7 of the 8 participating nurses supported continued use of the system, citing workflow efficiency gains.

**Conclusions:**

Miri-Alimi enabled patient-friendly PGHD collection without requiring log-ins or a dedicated app and demonstrated direct transfer of patient responses into the EMR. Its use was associated with effective transfer and structured integration of PGHD into the EMR, as well as high satisfaction among survey respondents and participating staff. Further studies should evaluate sustainability and associations with long-term clinical outcomes across diverse care settings.

## Introduction

The burden of cardiovascular diseases on the Korean health care system has steadily increased, with chronic cardiac conditions such as heart failure significantly contributing to this burden. Cardiovascular mortality in South Korea reached 123 per 100,000 persons in 2018, and hospitalizations due to circulatory system diseases increased by approximately 3.7-fold from 169,000 in 2002 to 630,000 in 2018 [1]. Furthermore, heart failure prevalence increased from 0.77% in 2002 to 2.53% in 2020 [2]. Patients with cardiovascular diseases commonly have multiple comorbidities, and this is particularly evident among patients with heart failure; for instance, 78.7% of patients with heart failure also had hypertension, 58.8% also had diabetes mellitus, and 15.8% also had chronic kidney disease [2].

Clinicians face ongoing challenges in managing patients with complex medical histories, such as the thorough collection and documentation of patient data, while considering diverse comorbidities. Effective management of chronic conditions requires continuous monitoring rather than intermittent isolated interventions [3]. Additionally, substantial discrepancies exist between the patient- and physician-based recognition of symptoms in routine clinical practice, potentially hindering precise assessment and appropriate clinical decision-making [4].

Many medical institutions have adopted electronic patient-reported outcomes (ePROs) to address these issues. A patient-reported outcome refers to any report on a patient’s health condition directly provided by the patient, thus shifting clinical consultations toward patient-centered care [5,6]. In a nonrandomized controlled study, Rocque et al [7] demonstrated that the implementation of an electronic patient-reported outcome–based remote symptom monitoring program in routine cancer care was associated with significantly lower hospitalization rates compared with historical controls, with a 19% reduction at 3 months (risk ratio 0.81; 95% CI, 0.73‐0.91) and a 13% reduction at 6 months (risk ratio 0.87; 95% CI, 0.80‐0.96). Patient-reported outcomes are typically collected through clinician-initiated tools embedded within health care systems, whereas patient-generated health data (PGHD) are produced outside clinical encounters using widely accessible commercial platforms such as mobile apps and wearable devices. PGHD are distinguished by 2 key characteristics: data capture is primarily patient driven rather than provider directed, and decisions regarding data sharing and distribution are controlled by patients, encompassing self-measured physiological and behavioral data collected through home devices, mobile apps, or wearable technologies [8,9]. PGHD enrich clinical information by providing personalized and contextual insights and promoting self-care practices among patients [10]. In oncology, PGHD supports survivorship care by fostering patients’ autonomy, improving health outcomes, and contributing to population-level health [11]. When designing ePRO systems, investigators decide whether patient-reported outcome measures should be collected directly within participating health systems or integrated from external platforms into individual electronic records, a choice that carries important implications for clinical usability and future research [12]. Despite the widely acknowledged significance of EHR integration, prior studies indicate that approximately 70% of PGHD implementations lack effective EHR linkage, which underscores the need for more streamlined mechanisms for data incorporation in future applications, particularly in cardiology clinics [13,14].

In response, we developed “Miri-Alimi,” a mobile previsit questionnaire system delivered via social messaging platforms commonly used in South Korea. Designed to lower digital barriers and facilitate integration with a hospital’s in-house automated electronic medical record (EMR) system, Miri-Alimi aims to enhance outpatient documentation, improve patient-clinician communication, and streamline clinical workflows. This study evaluated the implementation of Miri-Alimi in a cardiology outpatient clinic by assessing the participation rates, completeness of EMR documentation, and satisfaction among patients and health care providers.

## Methods

### Study Design and Population

**Figure 1.**
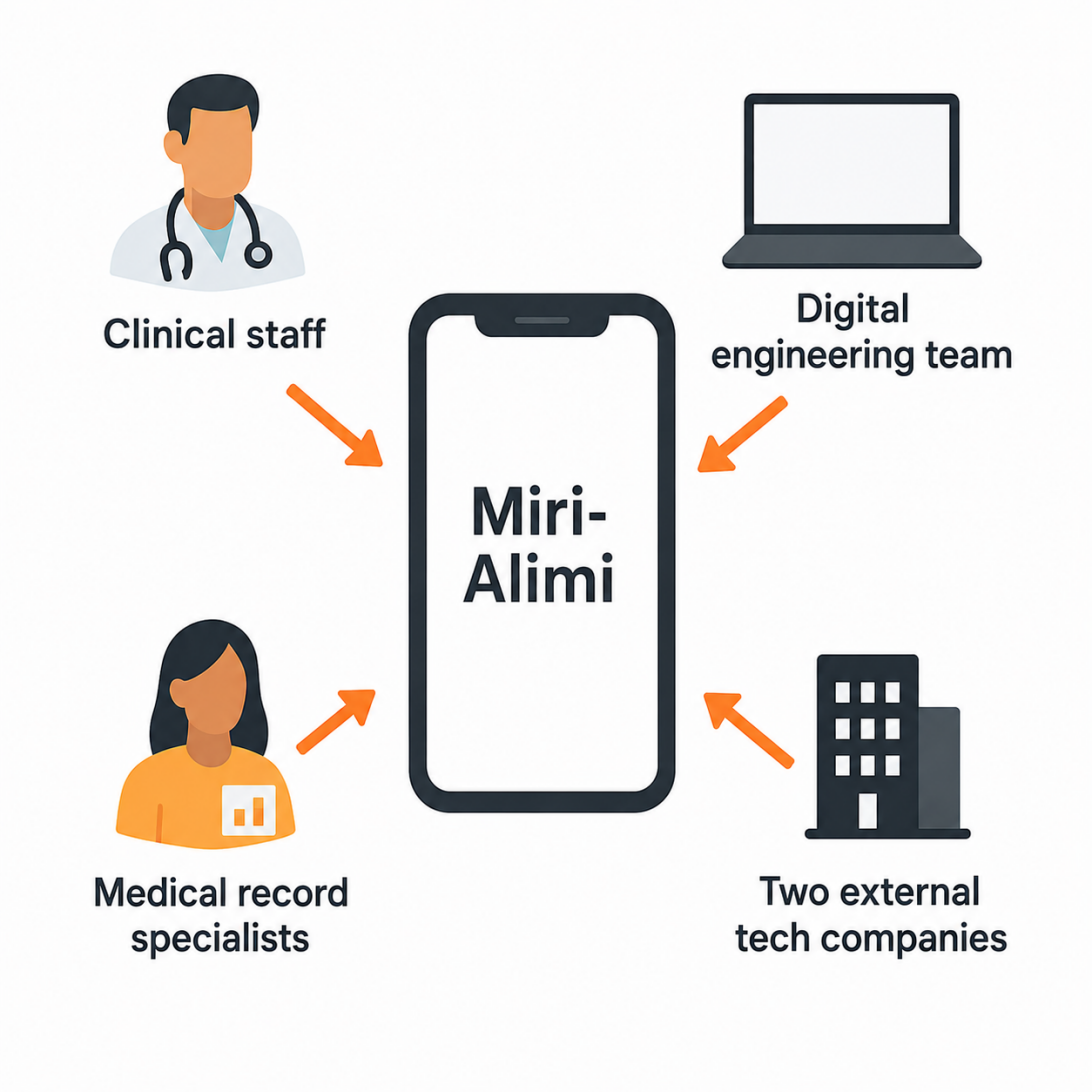
Multidisciplinary team–based development of Miri-Alimi, including clinical staff (questionnaire development, chart review, and efficiency evaluation), medical record specialists (target patient selection and EMR formatting), 2 external tech companies (system development and data transfer), and the digital engineering team (EMR management). EMR: electronic medical record.

This single-center prospective study was conducted at the outpatient cardiology clinic of a tertiary hospital. The digital service Miri-Alimi was developed through collaboration of a multidisciplinary team including clinical staff, medical record specialists, a digital engineering team, and 2 external technology companies responsible for message delivery and PGHD integration into EMRs (Figure 1). Miri-Alimi was officially launched following a 3-month pilot phase to enhance system reliability, user convenience, and readability.

A multidisciplinary team was composed of clinical staff who were in charge of developing questionnaires, reviewing patient charts, and evaluating the efficiency of Miri-Alimi; medical record specialists who were in charge of selecting target patients and formatting questionnaire results into electronic medical record; 2 external tech companies (Lemon Healthcare and LC Tech) who built and managed the mobile questionnaire system and were in charge of the transfer of survey data into the EMR; and the digital engineering team who managed errors in running the survey.

Patients were enrolled between August and November 2024 and categorized into 2 groups: new patients with cardiovascular disease (first-time visits for various cardiovascular conditions) and repeat patients with heart failure (follow-up visits for existing patients with heart failure). The eligibility criteria included an age of ≥18 years and the capability to use a mobile phone with messaging features. Patients without access to a mobile phone or those who were unable to complete the survey because of severe visual or cognitive impairment were excluded.

During the study period, 751 patients, comprising 282 new patients with cardiovascular disease and 469 repeat patients with heart failure, received invitations to use Miri-Alimi. Invitations were sent before each scheduled outpatient visit.

### Ethical Considerations

Ethical approval for human subject research was obtained from the Institutional Review Board of Korea University Guro Hospital (approval 2024GR0221). All participants provided informed consent electronically prior to participation. The consent included authorization for the collection of personal identifiers, medical information, and survey responses, as well as permission for secondary analysis without the need for additional consent. No financial or nonfinancial incentives were provided for participation.

All data exported from the Miri-Alimi platform were anonymized prior to analysis to protect participant confidentiality. Survey results were not accessible to patients through the mobile system after submission; access was restricted to authorized clinicians during face-to-face consultations or through formal, in-person medical record requests verified by hospital staff.

Patient authentication was conducted using birthdate verification, which was chosen to lower digital barriers and improve accessibility for patients, particularly older adults or those with limited digital literacy.

Patient responses were initially stored on Lemon Healthcare’s external cloud server and subsequently transferred in real time to the hospital’s designated external server, hosted by Naver Cloud (NAVER Corp), which maintains multiple security certifications. In compliance with the Korean Personal Information Protection Act (PIPA), all data outsourcing and cloud storage processes adhered to legal and regulatory requirements.

In accordance with Article 26 of PIPA, a formal data processing agreement was established, clearly specifying the purpose of data outsourcing, restrictions on further processing, technical and administrative safeguards, and the designation of Lemon Healthcare and Naver Cloud as authorized data processors.

Additionally, pursuant to Article 29, appropriate technical and managerial measures were implemented to ensure the protection of personal information. All server-client communications were encrypted using Secure Sockets Layer (SSL) and hypertext transfer protocol secure (HTTPS) protocols. User sessions were protected through JSON Web Token (JWT) authentication, with automatic termination after 30 minutes of inactivity or upon survey completion. Furthermore, an administrative dashboard enabled authorized clinical staff to monitor the delivery status of survey invitations, track real-time response completion, and review system-generated analytics to support quality control and data integrity throughout the study.

### Previsit Questionnaire Development

A multidisciplinary team of cardiologists, cardiovascular nurses, and medical information specialists created a Korean-language electronic questionnaire optimized for outpatient cardiovascular care. Questionnaire development proceeded through several multidisciplinary meetings, during which items were iteratively refined to minimize discrepancies in symptom interpretation between patients and clinicians and to ensure compatibility with outpatient workflows, particularly for heart failure care. A key design objective was the seamless transfer of patient-entered data into EMR fields aligned with established scales, including the New York Heart Association (NYHA) functional classification and Canadian Cardiovascular Society (CCS) angina grading. For example, a patient response of “I experience shortness of breath or fatigue during ordinary activities such as walking on level ground” was automatically mapped to NYHA class III within the EMR. This automated semantic mapping promotes objective and consistent documentation of symptom severity and facilitates longitudinal monitoring of clinical status. Survey-derived NYHA class was automatically populated in the EMR and could be modified by clinicians during the encounter. Two versions of the questionnaire were developed (Textboxes1  2) for 2 patient groups. The questionnaire for new patients was designed to systematically assess cardiovascular symptoms and to obtain detailed medical histories. The questionnaire for follow-up patients with heart failure was designed to capture essential clinical information regarding patient status, including general health condition, symptom severity, medication adherence, and use of medications prescribed outside the clinic.

Textbox 1. **The content of questionnaire for first-visit patients.**

- Symptoms and severity: dyspnea, chest pain, palpitations, syncope
- Comorbidities: hypertension, diabetes mellitus, dyslipidemia, coronary artery disease, arrhythmia, stroke
- Lifestyle and social history: drinking, smoking, exercise, occupation
- Family history
- Problems or questions for medical staff

Textbox 2. **The content of questionnaire for follow-up patients with heart failure.**

- General health rating (0-10)
- Symptoms and severity: dyspnea, chest pain, palpitation, dizziness, syncope
- Signs of decompensation: peripheral edema, weight change compared to last visit
- Medication from external providers
- Medication adherence assessment (≥80% doses taken) and barriers
- Home-measured vital signs (blood pressure and heart rate) and weight
- Dyspnea, chronic fatigue, and peripheral edema assessed (0-10)
- Problems or questions for medical staff

In the new patient questionnaire, thirteen stem questions (with conditional subitems) gathered baseline data on (1) cardiac symptoms, (2) past medical history and comorbidities, (3) social history, and (4) family history of cardiovascular disease.

In the heart failure follow-up questionnaire, seventeen stem questions were supplemented with validated scoring scales and interval changes and adherence were monitored. Patients (1) rated global health status, (2) graded dyspnea and chest pain, (3) reported changes in dyspnea since the previous visit, (4) reported peripheral edema or weight gain >3 kg, (5) detailed medication adherence (≥80% of prescribed doses taken) and listed drugs prescribed outside of the institution, and (6) entered home-measured vital signs and body weight.

Both instruments relied primarily on fixed-choice items, and the follow-up questionnaire included 6 short-answer fields. Each questionnaire ended with a free-text section on patient-identified concerns.

### Delivery of the Previsit Questionnaire

The Miri-Alimi platform was integrated into the hospital’s appointment scheduling system, enabling automated distribution of previsit surveys. Considering South Korea’s high smartphone penetration rate of 95.1% in 2024 [15], a personalized URL linking directly to the survey was delivered via KakaoTalk (versions 10.9.0 to 11.3.0; Kakao Corp), a messaging platform used by 98.9% of Korean mobile users [16], 3 days before the scheduled outpatient appointment. For patients without access to KakaoTalk, survey invitations were alternatively dispatched through multimedia messaging service (MMS).

**Figure 2.**
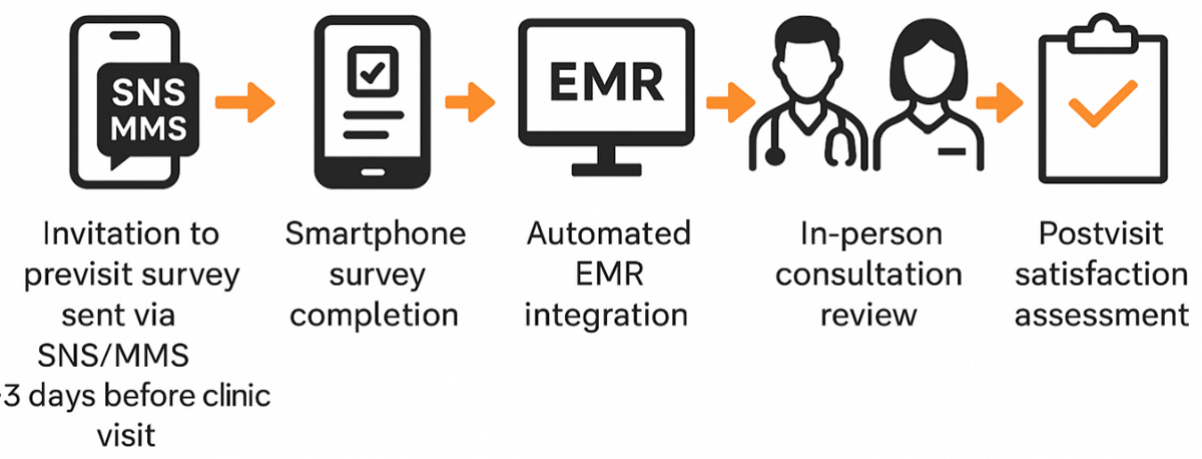
Workflow of this study EMR: electronic medical record; MMS: multimedia messaging service; SNS: social networking service.

The survey was designed for ease of access, requiring neither the installation of an app nor user authentication through username and password. Instead, identity verification was conducted by matching patient-entered birthdates with EMRs. Patients provided informed consent electronically regarding the collection and use of personal information for research purposes before proceeding with the survey. The survey URL remained accessible until the time of consultation, allowing patients to complete the questionnaire in the clinic waiting area if necessary. An automated reminder was dispatched 1 day before the appointment for those with incomplete responses. Each patient received only 1 questionnaire prior to their index visit during the study period (Figure 2).

The survey used a responsive HTML-based interface compatible with both desktop and mobile devices. Upon accessing the URL, patients encountered a dashboard displaying assigned surveys, which could be edited until the scheduled consultation. The survey used mandatory response fields to minimize missing data. Additionally, conditional branching logic was used to enhance the relevance and efficiency of the survey flow. Completed survey responses were transferred directly to the institution-specific EMR of Korea University Medical Center via Lemon Healthcare’s proprietary Quick Application Programming Interface (API) Builder, enabling immediate clinical use.

### Data Collection and EMR Integration

**Figure 3.**
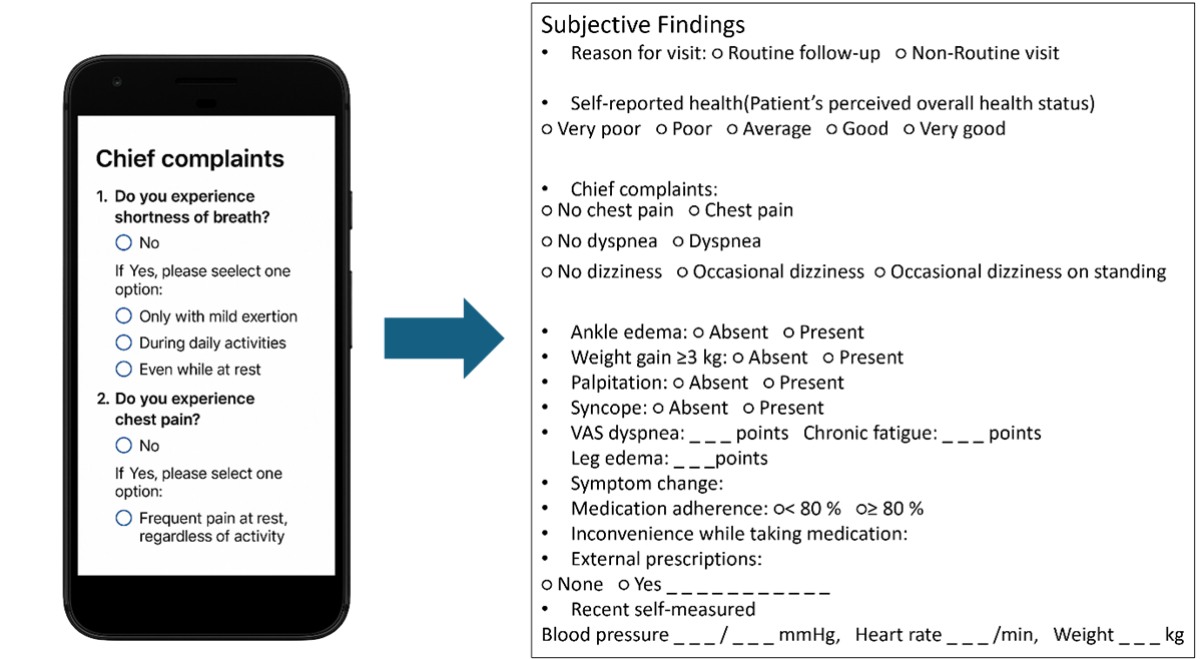
Illustration of the previsit smartphone survey (left) that was integrated into the electronic medical records (right) for patients with heart failure during follow-up visits. VAS: visual analog scale.

The completed survey responses were automatically transmitted to the hospital’s EMR system. Each survey item was mapped to a corresponding field in a structured clinical note, enabling standardized documentation. For example, self-reported symptom severity and general health status were incorporated into the review of systems section (Figure 3). At the time of the visit, the clinicians reviewed the patients’ responses transferred to electronic charts. If a patient did not complete the survey prior to the appointment, no previsit entry was generated, and routine in-person medical history interviews were conducted.

### Clinic Visit Workflow and Postvisit Surveys

During clinical consultations, physicians were encouraged to incorporate the information provided by patients through Miri-Alimi into their evaluations. After consultation, patients who responded to the survey received a postvisit satisfaction survey delivered through the same messaging system.

The patient satisfaction survey comprised 15 items. Among these, 13 items related to appropriateness, acceptability, and satisfaction were rated on a 5-point Likert scale (1=strongly disagree, 5=strongly agree). Of these, 2 items assessed the appropriateness of the PGHD collection method and number of survey questions. Three items evaluated acceptability: ease of understanding the questions, difficulty in providing responses, and overall usability of the system. Eight items measured satisfaction with the perceived usefulness of Miri-Alimi. Two items were open-ended; the first assessed the time required to complete the survey and the second allowed patients to provide feedback in a free-text space with unlimited word count.

Separately, all participating clinical staff (n=10; 2 cardiologists and 8 nurses) were invited to complete a provider survey after the study period. The staff questionnaire comprised 15 items for cardiologists and 8 items for nurses. These surveys included 13 and 6 Likert-scale items assessing system acceptability and satisfaction, respectively, 1 item evaluating perceived changes in consultation time, and 1 open-ended item for additional comments.

### Outcome Measures

The primary outcomes were the rates of previsit survey responses and EMR data completion. The response rate was defined as the proportion of eligible clinic visits for which the patient submitted a previsit questionnaire. The rates were calculated separately for new patients and for those with follow-up visits for heart failure.

Data completeness was evaluated using a predefined EMR completeness score applied to follow-up visits for heart failure. Follow-up patients with heart failure formed a clinically homogeneous population, which permitted prior specification of disease-specific documentation elements. Based on consensus between 2 participating cardiologists, 3 core documentation parameters relevant to routine heart failure care were selected: dyspnea status, presence of peripheral edema, and medication adherence status. Each parameter received 1 point when the corresponding information was explicitly documented in the EMR clinical note, either through automated transfer of questionnaire responses for responders or through routine clinician documentation for nonresponders. For medication adherence, documentation was considered complete when the clinical note explicitly indicated whether adherence was above or below the 80% threshold. The total EMR completeness score ranged from 0 to 3, with higher scores indicating more complete structured clinical documentation.

Completeness scores among responders were derived automatically from structured data generated by the previsit questionnaire. In contrast, completeness scores for nonresponders were determined through a comprehensive manual review of the EMRs performed by the study investigators. For nonresponders, a point was assigned only when the relevant parameter itself was explicitly documented.

All relevant components of the EMR were reviewed, including structured symptom fields, free-text clinical notes, and prescription records. Free-text entries that lacked explicit clinical definition or standardized symptom descriptors, including nonspecific phrases such as “so-so” or “no interval change,” that suggested overall stability or interval status were not regarded as valid documentation of heart failure–related symptoms. To minimize subjective interpretation by chart reviewers and to ensure methodological consistency, such entries were assigned a completeness score of 0.

Secondary outcomes included measures of the utility of the ePRO system, specifically the proportion of follow-up patients with heart failure who reported taking medications prescribed outside the heart failure clinic and the acquisition rate of free-text entries describing those medications. Additional secondary outcomes included patient satisfaction (defined as the proportion of respondents who agreed or strongly agreed with positive statements), provider satisfaction, and the perceived impact on clinical workflow.

As an exploratory workflow-related measure collected separately from the Miri-Alimi platform, consultation time was estimated using EMR time stamps. Specifically, the attending physician was asked to perform an initial save of the EMR at the start of the outpatient encounter and a final save at the end of the consultation, and the interval between these 2 time points was used as a proxy for consultation duration. Consultation time was not a prespecified study end point. However, because objective time stamp data had been collected for a subset of visits, these data were analyzed exploratorily to complement provider-reported assessments of workflow efficiency.

### Data Analysis

Analyses were conducted using R software (version 4.4.2; R Foundation for Statistical Computing). Response rates and item-specific satisfaction rates were calculated for both the patient and provider postvisit surveys. Free-text responses were qualitatively analyzed to identify recurring themes.

Because this study was designed as a real-world implementation study, no a priori sample size calculation was performed. The study population therefore consisted of a sample of all eligible consecutive patients during the prespecified study period.

Descriptive statistics were used to summarize the patient demographics, clinical characteristics, and survey responses. Continuous variables are presented as means with SDs or medians with IQRs and were compared using 2-tailed *t* tests or Wilcoxon rank sum tests, as appropriate. Categorical variables are reported as frequencies and percentages and were compared using the chi-square test or Fisher exact test. Statistical tests were 2-tailed, with the significance level set at *P*<.05.

Exploratory analyses of consultation time were performed only for visits with available time stamp data. Given the substantial and differential missingness in consultation time data, formal statistical testing was not performed. Additional subgroup analyses were performed separately for first-visit patients and follow-up patients with heart failure. Given the substantial proportion of missing time stamp data, particularly among nonresponders, these analyses were considered exploratory and were interpreted with caution.

Differences in EMR completeness between responders and nonresponders were evaluated using Firth penalized logistic regression because the distribution of EMR completeness scores demonstrated substantial separation. Two prespecified binary outcomes were examined: complete documentation (score 3 vs scores 0‐2) in the primary analysis and higher documentation completeness (scores ≥2 vs scores 0‐1) in the supplementary analysis. To account for potential demographic confounding, particularly age-related differences in digital health engagement, both age and sex were included in the adjusted models.

## Results

### Patient Participation and Characteristics

**Figure 4.**
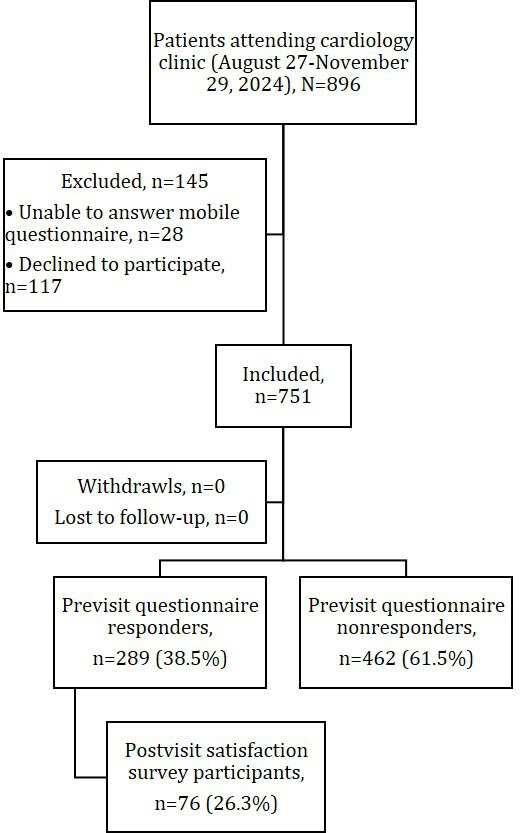
Study flow diagram.

**Figure 5.**
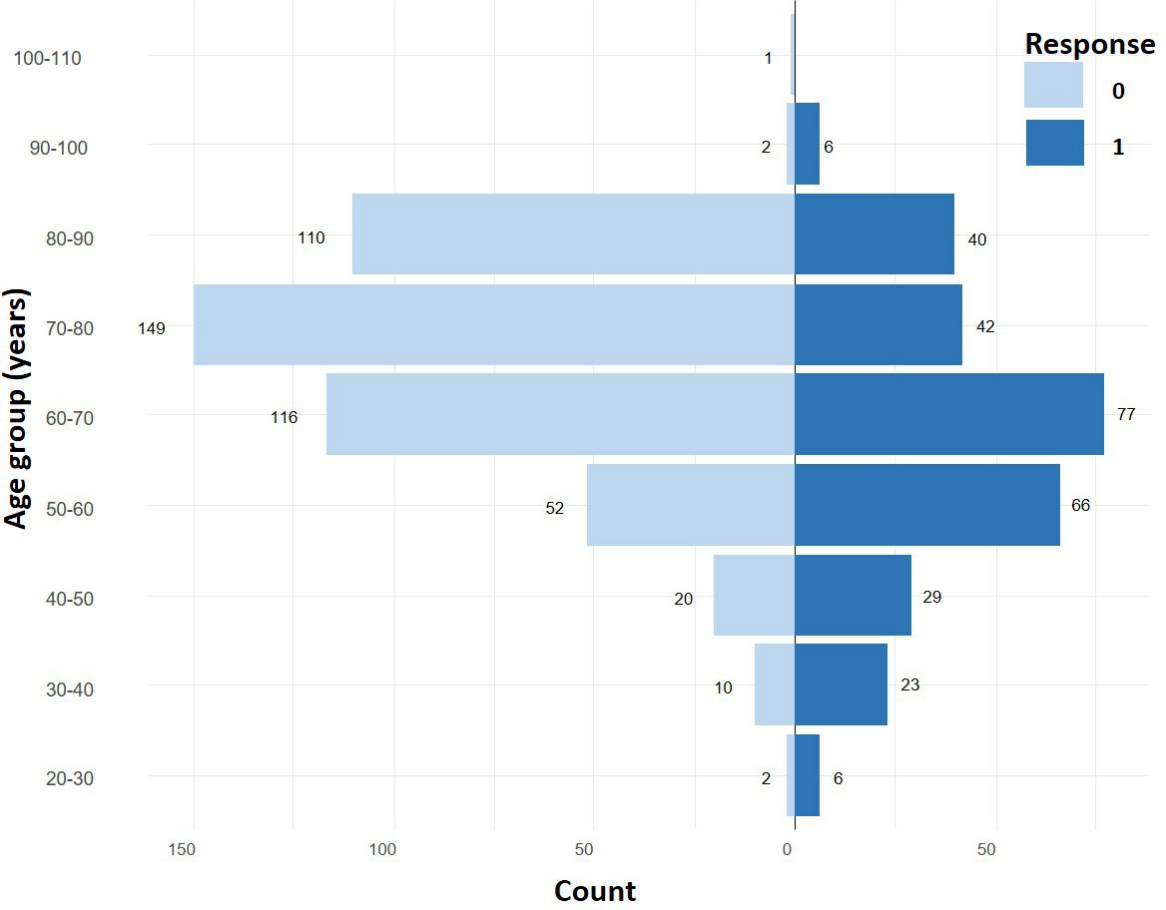
Histogram of age distribution among patients who completed the previsit survey (response=1) vs those who did not (response=0).

Of the 751 patients invited, 289 (38.5%) responded to the previsit questionnaire (Figure 4 and MultimediaAppendices 1  2). The response rates were 48.9% (138/282) among new patients and 32.2% (151/469) among follow-up patients with heart failure. The proportion of women was slightly higher among responders (147/289, 50.9%) than among nonresponders (194/462, 42%; *P*=.020). Moreover, responders were significantly younger than nonresponders (mean 62.0, SD 15.7 years vs mean 69.8, SD 12.5 years; *P<*.001; Table 1). Age distribution analysis showed lower response rates among patients aged ≥70 years, who constituted 56.7% (262/462) of nonresponders versus 30.4% (88/289) of responders (Figure 5). Patient participation was robust, with consistently high response rates exceeding 90% for all multiple-choice items. Nonconditional short-answer items achieved response rates exceeding 50%, while lower response rates were observed for items intended to be answered only when applicable (Table S2 in Multimedia Appendix 3).

**Table 1.** Comparison between responders and nonresponders for the 751 invited participants.

	Responders (n=289)	Nonresponders (n=462)	*P* value
New patients (n=282), n (%)	138/282 (48.9)	144/282 (51.1)	—^a^
Follow-up patients with HF^b^ (n=469), n (%)	151/469 (32.2)	318/469 (67.8)	—
Women, n (%)	147/289 (50.9)	194/462 (42)	.020
Age in years, mean (SD)	62.0 (15.7)	69.8 (12.5)	<.001

a Not applicable.

b HF: heart failure.

### EMR Documentation Completeness

**Figure 6.**
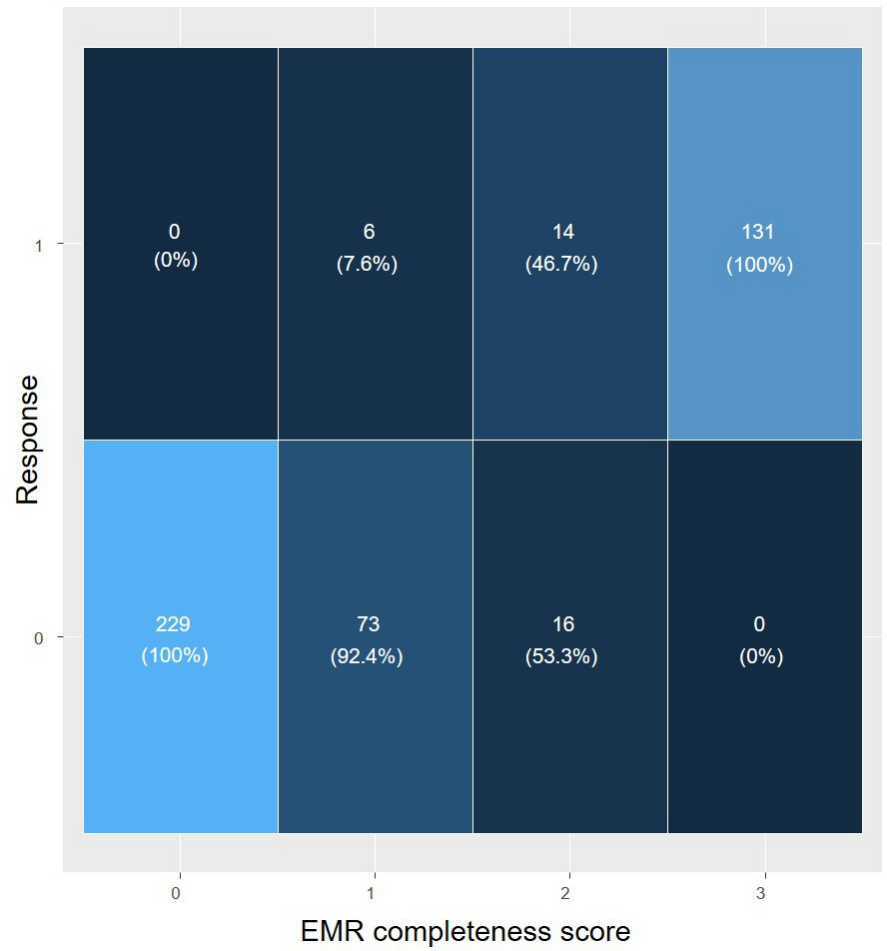
Cross-tabulated distribution of electronic medical record (EMR) completeness scores (0‐3) by previsit survey response status (0=nonresponder, 1=responder).

Among the 469 follow-up patients with heart failure, the completeness of the EMR documentation was significantly greater among those who completed the previsit survey. Responders (n=151) had a median EMR score of 3 (IQR 3‐3), reflecting complete documentation of dyspnea, peripheral edema, and medication adherence. In contrast, nonresponders (n=318) had a median EMR score of 0 (IQR 0‐1; *P<*.001), with only 28% (89/318) having any of the 3 parameters recorded (Table 2). The distribution of EMR completeness scores showed sparse outcome strata with quasi-complete separation (Figure 6).

**Table 2.** Data completeness of follow-up visits for patients with heart failure.

	Responders n=151	Nonresponders n=318	*P* value
Data completeness^a^ median (IQR) EMR score	3 (3-3)	0 (0‐1)	<.001

a Data completeness: composite EMR score ranging from 0 to 3, assigning 1 point each for explicit documentation of dyspnea status, presence of peripheral edema, and medication adherence status in the EMR. For medication adherence, documentation was considered complete when the clinical note explicitly indicated whether adherence was above or below the 80% threshold. EMR: electronic medical record.

### Descriptive Results of ePRO-Based Medication Reporting

Among 151 follow-up patients with heart failure who completed the previsit survey, 89.4% (135/151) responded to the question regarding the use of medications prescribed outside the heart failure clinic. Of these respondents, 53.3% (72/135) reported taking at least 1 medication not prescribed by the heart failure clinic. Of those reporting externally prescribed medications, 80.6% (58/72) provided specific medication details through the free-text input field (Table 3).

**Table 3.** Descriptive results of ePRO^a^-based medication reporting among follow-up patients with heart failure.

	Participants, n/N (%)
Response to question on medications prescribed outside the heart failure clinic	135/151 (89.4)
Reported use of medications prescribed outside the heart failure clinic	72/135 (53.3)
Completion of free-text medication entry among those reporting externally prescribed medications	58/72 (80.6)

a ePRO: electronic patient-reported outcome

### Patient Satisfaction

Of the 289 patients who completed the previsit questionnaire, 26.3% (76/289) participated in the postvisit satisfaction survey (Table 4 and MultimediaAppendices 1  2). The mean age of these respondents was 61.6 (SD 14.3) years; 42.1% (32/76) of the patients were women, and 42.1% (32/76) were new patients. Most respondents (60/76, 78.9%) reported that they completed the previsit survey within 10 minutes, with the majority (72/76, 94.7%) reporting that they completed it within 20 minutes (Table 5).

**Table 4.** Postvisit satisfaction survey.

	Participants, n/N (%)			
Participation rate	76/289 (26.3)			
First-visit patients	32/76 (42.1)			
Follow-up patients	44/76 (57.9)			
Women	32/76 (42.1)			

**Table 5.** Reported time required for previsit survey.

Time required for previsit survey, minutes	Participants, n (%)		
	Combined (n=76)	New patients (n=32)	Follow-up patients (n=44)
<10	60 (78.9)	23 (71.9)	37 (84.1)
10‐20	12 (15.8)	7 (21.9)	5 (11.4)
20‐30	2 (2.6)	1 (3.1)	1 (2.3)
>30	2 (2.6)	1 (3.1)	1 (2.3)

All 76 patients completed the 15-item satisfaction survey (Table S3 in Multimedia Appendix 3), and their satisfaction responses were overwhelmingly positive. Agreement rates for the 13 key items ranged from 82.9% (63/76) to 92.1% (70/76), each surpassing 70% (*χ*²_1_=4.8‐17.7, all *P*<.028 after false discovery rate adjustment). Item means were between 4.09 and 4.30 on the 5-point Likert scale, all exceeding the neutral midpoint of 3 (*t*_75_=12.4‐16.4, *P*<.001 with Bonferroni correction; Cohen *d*=1.5‐1.9), which confirms pronounced patient satisfaction with Miri-Alimi.

Among 76 participants, 92.1% (n=70) agreed that KakaoTalk or text messages were a suitable notification method, and 88.2% (n=67) found the number of questions appropriate. In terms of acceptability, 89.5% (68/76) reported that the questions were easy to understand, 86.8% (66/76) were able to complete the survey without assistance, and 85.5% (65/76) found it overall easy to use. Regarding perceived usefulness, 86.8% (66/76) to 89.5% (68/76) agreed that the survey was helpful in preparing for the visit and in communicating symptoms and history, while 82.9% (63/76) to 84.2% (64/76) found it beneficial for clinicians’ understanding and overall care (Table S3 in Multimedia Appendix 3). Overall satisfaction was reported by 85.5% (65/76), with 92.1% (70/76) expressing willingness to continue using the system and 84.2% (64/76) indicating they would recommend it to others.

### Provider Feedback

Feedback from ten clinical staff members (2 cardiologists and 8 nurses) supported the system’s utility. Nurse respondents (n=8) reported high agreement (88%) across domains, including question appropriateness, clinical utility, workflow efficiency, and satisfaction with continued use (Table 6). Aligning with Miri-Alimi’s goal of streamlining outpatient workflow, nurses reported that the system facilitated patient assessment and reduced both previsit preparation and in-clinic encounter times by allowing them to focus on key information rather than conducting an extensive history. Both cardiologists (n=2) endorsed the integration of Miri-Alimi into the clinical workflow and noted improvements in documentation and patient engagement, although only 1 reported a perceived reduction in in-clinic history-taking items (Table S5 in Multimedia Appendix 3).

**Table 6.** Postvisit satisfaction survey results of nurses (n=8).

Category	Item	Participants who agreed or strongly agreed, n (%)
Appropriateness	Appropriateness of number and types of questions	7 (87.5)
Clinical utility	Helpfulness of patient self-reporting in identifying patients	7 (87.5)
	Easier patient management compared to without Miri-Alimi	7 (87.5)
Workflow	Reduction in previsit preparation time	7 (87.5)
	Reduction in patient encounter time	7 (87.5)
Overall satisfaction	Overall satisfaction with care using Miri-Alimi	7 (87.5)
	Agreement on continued use and expansion of Miri-Alimi	7 (87.5)

### Exploratory Objective Consultation Time Data

Objective consultation time data were collected separately from the Miri-Alimi platform by measuring the interval between the physician’s initial EMR save at the start of the encounter and the final save at the end of the visit. However, these data were incomplete because of transmission errors and missed final saves during routine care. Of the 751 enrolled patients, consultation time data were available for 400 patients (Multimedia Appendix 4). Consultation time data were available for 208 of 289 (72%) responders and 192 of 462 (41.6%) nonresponders, respectively.

In the overall cohort with available time stamp data, consultation time, measured in seconds, was descriptively longer among responders than among nonresponders (median 292, IQR 207-410 seconds vs median 250, IQR 182-358 seconds). In -subgroup analyses, consultation time did not show a clear descriptive difference between responders and nonresponders among follow-up patients with heart failure (100/151, 66.2% vs 119/318, 37.4%; median 294, IQR 218-380 seconds vs median 276, IQR 186-362 seconds). In contrast, among first-visit patients, consultation time was descriptively longer for responders than for nonresponders (108/138, 78.3% vs 73/144, 50.7%; median 288, IQR 201-456 seconds vs median 216, IQR 179-333 seconds). However, these comparisons are descriptive only and do not imply statistical significance due to substantial and differential missingness in time stamp data.

### Firth Penalized Logistic Regression

In the Firth penalized logistic regression, survey response status was strongly associated with complete EMR documentation after adjustment for age and sex (adjusted odds ratio [OR] 3611.09, 95% CI 497.9‐459754.6; *P*<.001). In the sensitivity analysis using a less stringent threshold of EMR completeness (score ≥2 vs scores 0‐1), the association remained significant (adjusted OR 368.89, 95% CI 157.6‐1010.9; *P*<.001). Sex and age were not significantly associated with either outcome.

## Discussion

### Main Findings and Comparison With Prior Work

This nonrandomized service implementation study suggests that Miri-Alimi, a mobile-based previsit questionnaire system, is feasible for implementation in routine cardiology practice and was acceptable among responding patients and participating health care providers. The system improved the structured documentation of essential clinical data relevant to patient status assessment. In addition, PGHD collection and use were associated with favorable satisfaction among postvisit survey respondents.

However, previous studies investigating whether PGHD collection enhances clinical outcomes beyond satisfaction have yielded inconsistent results. Sandhu et al [17] observed no significant changes in heart failure quality of life scores, despite patients reporting that clinicians had an improved understanding of their symptoms. In contrast, Yamashita et al [18], in a randomized study, found significant enhancements in patient satisfaction and the quality of information provided by clinicians when using ePROs compared to traditional methods. These benefits were attributed primarily to more frequent patient follow-ups. Furthermore, Sandhu et al [17] highlighted that automated clinician alerts for clinically meaningful symptom deterioration could enhance the effectiveness of routine PGHD monitoring. Collectively, these insights emphasize that collecting PGHD data alone is insufficient and that timely reviews and responsive clinical actions are critical.

To facilitate wider adoption of PGHD collection and use in clinical settings, the process of notifying patients to submit PGHD and authenticating users to access PGHD platforms must be simplified. Traditionally, PGHD collection has relied on patient portals or dedicated apps, with notifications delivered via portal messages, emails, text messages, or in-clinic tablets [19,20]. Log-in requirements or app installations were identified in 16 of the 17 comparable programs reviewed by Austin et al [13], likely contributing to limited adoption. Prior studies reported low completion rates (6%‐10%) for email- or text-delivered portal surveys and moderate completion rates (up to 52%) for in-clinic tablet deployments, although these required human assistance (Table 7) [21-23]. While Yedulla et al [21] achieved higher completion rates (email: 52%, portal: 49%, control: 30%), their cohort was relatively young (mean age: 56 years) and restricted to participants with dual digital credentials, potentially excluding older adults and individuals with limited digital literacy. Consequently, selecting simple and widely accessible methods for patient invitation and authentication is essential to minimize biases related to age and digital barriers and promote broader patient engagement.

**Table 7.** Comparison of PGHD^a^ collection platforms regarding invitation method, access process, and EMR^b^ integration.

Authors	Country	Year	Title	Objective	Invitation method	Survey platform	EMR auto-integration	Participation rate
Miri-Alimi	Korea	2024	—^c^	—	SNS^d^ or MMS^e^	Website (no log-in required)	Yes	38.5%
Kouri et al [20]	Canada	2020	Primary care pre-visit electronic patient questionnaire for asthma: uptake analysis and predictor modeling	The objective of this study was to determine which factors influence the uptake and successful completion of the tablet questionnaire by analyzing its implementation in a primary care setting.	In-clinic invitation by an assistant	App in a tablet PC	No; manually recorded in a computer file by an assistant	52% uptake, 88% completed
Holt et al [22]	USA^f^	2021	Impact of pre-visit contextual data collection on patient-physician communication and patient activation: a randomized trial	The objective of this study was to determine if a digital tool designed to collect and present previsit contextual data improves communication and patient activation; secondarily, evaluate if use impacts outcomes by race	Email or postcard	Patient portal (log-in required)	Yes	30% (with outreach), 6% (email only)
Shucard et al [23]	USA	2022	Clinical use of an electronic pre-visit questionnaire soliciting patient visit goals and interim history: a retrospective comparison between safety-net and non-safety-net clinics	This study examined an initial step toward cogeneration of clinic notes by inviting patients to complete a previsit questionnaire that could be inserted into clinic notes by providers and describe the experience in a safety-net and non–safety-net clinic.	Email	Patient portal (log-in required)	Yes	3% (safety-net) vs 10% (non–safety-net)
Yedulla et al [21]	USA	2022	Pre-visit digital messaging improves patient-reported outcome measure participation prior to the orthopedic ambulatory visit	The purpose of this study was to determine the efficacy of 3 different methods with respect to previsit electronic patient-reported outcome measure completion.	Email or portal notifications	Patient portal (log-in required)	Yes	49% (portal), 52% (email), 30% (control)
Katzel et al [24]	USA	2023	Real-world use of electronic patient-reported outcome (ePRO) tools integrated in the electronic medical record during radiation therapy for head and neck cancer: feasibility study	The aims of the study were to assess the feasibility of ePROs integrated within the EMR during radiation therapy for patients with head and neck cancer within a large health care delivery system and to evaluate its resource use and practitioner assessment.	Email	Patient portal (log-in required)	Yes	34 patients enrolled, 194 ePRO surveys completed
Hassett et al [25]	USA	2022	eSyM: an electronic health record-integrated patient-reported outcomes-based cancer symptom management program used by six diverse health systems	The objective of this study is to create and deploy an electronic health record (EHR)–integrated patient-reported outcome (PRO)–based symptom management program as part of routine clinical practice across multiple health systems.	Portal notifications	Patient portal (log-in required)	Yes	In total, 3352 patients completed 26,268 symptom questionnaires between September 2019 and the study cut-off date.
Yoon et al [26]	Korea	2024	Effectiveness of a smartphone app–based intervention with Bluetooth-connected monitoring devices and a feedback system in heart failure (SMART-HF Trial): randomized controlled trial	The study team developed a mobile health platform for heart failure self-care to evaluate whether a smartphone app–based intervention with Bluetooth-connected monitoring devices and a feedback system can help improve heart failure symptoms.	Portal notifications, Bluetooth-connected feedback	Mobile app (log-in required)	No	Patients who did not use the app at least once were excluded. In total, 77 patients (39 and 38 in the intervention and control groups, respectively) were included.

a PGHD: patient-generated health data.

b EMR: electronic medical record.

c Not applicable.

d SNS: social networking service.

e MMS: multimedia messaging service.

f USA: United States.

Another major challenge in PGHD use is seamless EMR integration. Accessing separate PGHD platforms and manually organizing and inputting data increase the workload of clinicians [19]. Austin et al [13] reported that manual transcription of PGHD was necessary in 35% of the programs reviewed. Reading et al [27] further highlighted the substantial need for the electronic integration of PGHD into existing clinical systems. Even in US clinics where EMR systems automatically upload PGHD, patient log-ins via embedded portals remain necessary [24,25]. Similarly, a recent Korean prototype collected PGHD in a standalone system without EMR integration (Table 7) [26]. Thus, achieving both simplified patient access and seamless EMR integration remains challenging. The Miri-Alimi system addresses these issues through direct EMR integration, thereby significantly reducing the clinician burden.

### Strengths and Limitations

Miri-Alimi distinguishes itself by addressing the major challenges in PGHD collection through a collaborative, multidisciplinary approach. First, it facilitates high patient participation by eliminating complex portal access procedures and distributing survey links via widely used messaging platforms (KakaoTalk and MMS). Leveraging this approach, Miri-Alimi achieved an overall response rate of 38%, substantially outperforming email-based portals.

Second, iterative pilot testing enabled the development of comprehensive yet user-friendly questionnaires tailored specifically for patients with cardiovascular diseases. These questionnaires were rigorously evaluated by a multidisciplinary team to ensure that the patients could complete them independently. Consequently, over 80% of respondents reported satisfaction with the questionnaire delivery method, question quantity, and ease of understanding. Incorporating standardized symptom scales (NYHA and CCS) enabled patients to effectively quantify symptom severity. Responders had significantly improved documentation of symptoms such as dyspnea, edema, and medication adherence in the EMR compared with nonresponders, demonstrating Miri-Alimi’s ability to accurately capture clinically relevant data, thus reducing information loss and discrepancies between patient and clinician perceptions.

Third, Miri-Alimi significantly reduced the workload of the medical staff by eliminating the barriers associated with accessing separate PGHD platforms and manually entering data into EMRs. It streamlined the clinical workflow by enabling patients to document their medical histories prior to visits, enhancing clinicians’ understanding, and improving patient-clinician communication.

Another strength of this study is the potential generalizability of the proposed PGHD-EHR integration framework. Survey responses were linked to the hospital EHR through a patient identifier–based matching process, without requiring modification of the core clinical system. Many hospitals already operate automated MMS- or SNS-based outpatient notification systems. Use of these existing communication channels, together with a PGHD platform that supports identifier-based linkage and standardized data export, allows the proposed workflow to be implemented across heterogeneous EHR environments with minimal structural change.

Nonetheless, this study has several limitations. First, it was conducted at a single tertiary hospital in South Korea using Korean-language questionnaires and communication tools tailored to the local health care context. Accordingly, the generalizability of the findings to other countries or health care systems may be limited.

Second, the primary outcome of documentation completeness was evaluated only in follow-up patients with heart failure, a clinically homogeneous subgroup in which disease-specific documentation elements could be prespecified and assessed consistently. This design substantially narrows the scope of the study’s conclusions, as the core metric of success was validated only within this specific subgroup rather than across the broader cardiovascular outpatient population, including patients with newly diagnosed cardiovascular disease. Moreover, the very large ORs and wide CIs indicate substantial imbalance in score distributions between groups. These estimates therefore support the direction of the association but not its precise magnitude.

Third, the system specifically targeted smartphone users, thereby excluding individuals with physical or cognitive impairments that limited completion of mobile questionnaires, as well as those lacking smartphones or internet access. Although the platform was intentionally designed to minimize digital barriers by allowing access through simple invitation links without log-in or app installation, important disparities in participation persisted. Responders were significantly younger than nonresponders, and response rates were notably lower among patients aged 70 years and older. These findings suggest that even simplified mobile-based PGHD systems may underrepresent elderly patients and exacerbate disparities in care. This concern is particularly important in heart failure clinics, where older adults constitute a large proportion of the patient population and frequently carry the highest disease burden. Accordingly, mobile PGHD platforms may serve as useful adjunctive tools in clinical practice; however, careful attention to patients who may be marginalized in the clinical setting should continue to be maintained during in-person care. Moreover, this study is subject to residual confounding due to unmeasured variables relevant to digital health interventions. Factors such as socioeconomic status, digital literacy, educational attainment, and baseline health status were not captured in the analysis and may have independently influenced both the likelihood of questionnaire completion and the extent and quality of mobile documentation.

Fourth, the absence of prior sample size calculation is an important limitation of this study. The sample comprised all eligible consecutive patients willing to participate during the study period, and the final sample size was therefore determined pragmatically rather than statistically.

Fifth, the use of birthdate-only verification constitutes a significant security vulnerability. Because access to the PGHD portal required only the patient-specific invitation link and the patient’s birthdate, any individual who obtained both could theoretically submit inaccurate or false patient-reported information into the EMR. Although clinicians reviewed the structured PGHD responses during the outpatient encounter and could revise the medical record after consultation when necessary to better reflect the patient’s actual condition, this review process does not mitigate the fundamental vulnerability of the authentication design. Accordingly, future implementations will require stronger security measures that reduce the risk of unauthorized access or inaccurate data entry.

Sixth, the automatic mapping of patient-reported symptoms to NYHA functional class without initial clinician adjudication may introduce misclassification and potential anchoring bias relative to clinician-determined functional assessment. Although survey response options were developed through iterative discussions among cardiologists and medical information specialists to translate NYHA functional classes into patient-friendly language, discrepancies between survey-derived and clinically adjudicated NYHA classification may still occur.

Seventh, another methodological limitation concerns the primary outcome of EMR completeness. The completeness score compared 2 inherently different documentation processes: automatically structured EMR fields among responders versus routine clinician documentation among nonresponders. Because the intervention directly transferred prespecified questionnaire items into the EMR, the completeness metric likely favored structured documentation. Therefore, the higher EMR completeness observed among responders should be interpreted primarily as improved structured capture and transfer of patient-reported information, rather than as proof that the intervention improved the overall quality of clinical care.

Eighth, a limitation pertains to the dichotomization of the ordinal EMR completeness score in the regression analysis. To evaluate the association between survey response and EMR completeness, Firth penalized logistic regression was used to address quasi-complete separation. In this context, the original ordinal EMR completeness score of 0 to 3 was dichotomized into ≥2 versus 0 to 1. Although this approach facilitated model convergence, it may have led to loss of information and reduced statistical efficiency, thereby limiting the ability to detect graded differences across levels of documentation completeness.

Ninth, an additional limitation relates to the objective assessment of workflow efficiency. Although consultation time data were collected for a subset of visits, these data were incompletely captured and were susceptible to operational error. Consultation duration was defined as the interval between the physician’s initial and final EMR entries, a measure inherently dependent on clinician behavior and therefore prone to substantial missingness. Missing time stamp data were differentially distributed between responders and nonresponders and were influenced by workflow-related factors, suggesting a missing-not-at-random mechanism. Consequently, the observed differences in median consultation time between groups may not reflect clinically meaningful differences in real-world practice, and this study does not establish whether the use of Miri-Alimi altered overall consultation duration. Furthermore, because only total consultation time was available, the study could not evaluate how time was distributed within the clinical encounter. Accordingly, any interpretation that time saved from routine history taking was reallocated to other components of the visit, such as addressing patient concerns or performing physical examination, remains hypothetical and was not directly assessed. Therefore, all analyses of consultation time should be considered exploratory and hypothesis generating.

Tenth, the operational value of PGHD-supported care should not be defined solely by whether the overall visit becomes shorter. Even if previsit PGHD reduces time spent on routine history taking, the time saved may instead be redirected toward clarification of patient concerns and more focused clinician-patient communication. In this context, longer consultation time among responders may reflect redistribution of clinical attention rather than inefficiency. Future studies should therefore incorporate prospectively defined and automatically captured workflow metrics, including time spent on routine history taking, total encounter duration, and postvisit charting burden, to better quantify the operational impact of PGHD-enabled care.

Eleventh, the number of participating clinicians was small, which limited the statistical power for analyses of provider satisfaction. Larger, multicenter studies involving more diverse clinical staff will be required to validate these findings.

Furthermore, although the Miri-Alimi questionnaires were developed through expert consensus and iterative multidisciplinary pilot testing, the instruments did not undergo formal psychometric validation. Specifically, internal consistency, content and construct validity, criterion validity, and test-retest reliability were not formally evaluated. Accordingly, this study should be interpreted primarily as a feasibility and implementation study of a PGHD-integrated questionnaire system rather than as a formal validation study of a patient-reported outcome instrument.

Additionally, medication adherence was assessed using a single, nonvalidated binary item. Given that medication adherence is a multidimensional construct encompassing both intentional and unintentional nonadherence, use of validated or prescription-based measures (eg, guideline adherence indices) would have enabled more detailed characterization of adherence patterns, including reasons for missed or discontinued medications.

Moreover, the patient-reported external medication feature did not fully realize its intended clinical utility for medication reconciliation. Among patients who reported taking medications prescribed outside the heart failure clinic, 81% provided additional details through the free-text field. However, these entries often relied on nonspecific terminology rather than precise drug or ingredient names. Consequently, although the responses were useful for identifying the presence of externally prescribed medications and for capturing clinically relevant categories that could affect decision-making, such as newly added medications and the use of anticoagulants, antiplatelet agents, and diuretics, they were insufficient for accurate medication-level reconciliation. This feature should therefore be regarded as providing partial contextual support rather than comprehensive reconciliation. To overcome this limitation, system enhancements incorporating linkage with national health insurance claims–based drug utilization review (DUR) data are currently under development and may allow more accurate integration of externally prescribed medication information into the EMR.

Furthermore, the limited number of patients (n=76) and clinical staff participating in the postvisit surveys may constrain the representativeness of the study population and introduce potential selection bias. The small number of participating clinicians also limited the statistical power of analyses related to provider satisfaction. These results should be interpreted as descriptive perceptions from a highly selected subgroup rather than as definitive evidence of overall system acceptability. These limitations reflect the study design, which evaluated a single outpatient encounter at a single cardiovascular center and therefore did not permit assessment of longitudinal participation or sustained use of the Miri-Alimi system. As the project is extended over time and expanded to include multiple centers, increased patient enrollment and broader staff participation may mitigate these limitations and enhance external validity.

Finally, another important limitation is that the study did not evaluate actual changes in patient management or clinical outcomes. Although key parameters relevant to patient status were successfully captured through the PGHD platform and incorporated into the EMR, the downstream impact on clinical care was not examined. We did not assess whether these data prompted timely medication adjustments, escalation of care, or other management decisions. In addition, clinical end points such as hospital readmission, emergency department use, and survival were not evaluated. Therefore, this study demonstrates the successful documentation and EMR integration of selected PGHD but does not establish whether such integration translates into improved clinical outcomes. Future studies are warranted to determine whether EMR integration of these PGHD leads to more timely therapeutic adjustment, fewer hospital readmissions, or lower cardiovascular mortality across diverse care settings.

### Conclusion

Miri-Alimi, a mobile-based platform designed to collect PGHD and integrate the data directly into the EMR, demonstrated feasibility and acceptability in routine cardiology outpatient practice. By leveraging widely used messaging services and eliminating barriers such as log-in or app installation, the system achieved patient participation of 38.5% (289/751). In follow-up patients with heart failure, documentation of key clinical parameters—dyspnea, peripheral edema, and medication adherence—was significantly more complete among responders (median score 3, IQR 3‐3) than among nonresponders (median score 0, IQR 0‐1; *P*<.001). Among patients who completed both the previsit questionnaire and the postvisit satisfaction survey, 82.9% (63/76) to 92.1% (70/76) agreed that the system was appropriate, user-friendly, and beneficial to their care experience. In addition, both participating cardiologists and 7 of 8 participating nurses endorsed continued use of the system, noting perceived improvements in workflow efficiency.

## Supplementary material

10.2196/81317 Multimedia Appendix 1 Dataset of previsit and postvisit survey responses of new cardiovascular patients.

10.2196/81317 Multimedia Appendix 2 Dataset of previsit and postvisit survey responses of follow-up patients with heart failure.

10.2196/81317 Multimedia Appendix 3 A previsit survey screen displaying patient-friendly, electronic medical record screen with previsit survey responses integrated; previsit questionnaire for first-visit cardiovascular patients; previsit questionnaire for follow-up patients with heart failure; postvisit satisfaction survey for patients; postvisit satisfaction survey for nurses; and postvisit satisfaction survey for cardiologists.

10.2196/81317 Multimedia Appendix 4 Patient consultation duration.

## Abbreviations

CCS: Canadian Cardiovascular Society
DUR: drug utilization review
EHR: electronic health record
EMR: electronic medical record
ePRO: electronic patient-reported outcome
HTTPS: hypertext transfer protocol secure
JWT: JSON Web Token
MMS: multimedia messaging service
NYHA: New York Heart Association
PGHD: patient-generated health data
SNS: social networking service
SSL: Secure Sockets Layer
