# Supplementary material for: Implementation and Evaluation of a Social Networking Service–Based Mobile Patient-Generated Health Data System With Direct Electronic Medical Record Integration: Prospective Observational Study
Source: JMIR Med Inform. 2026 Jun 23;14:e81317. doi: 10.2196/81317 (PMC13290106; doi:10.2196/81317)
Supplement: Multimedia Appendix 3 [file medinform-v14-e81317-s003.docx]

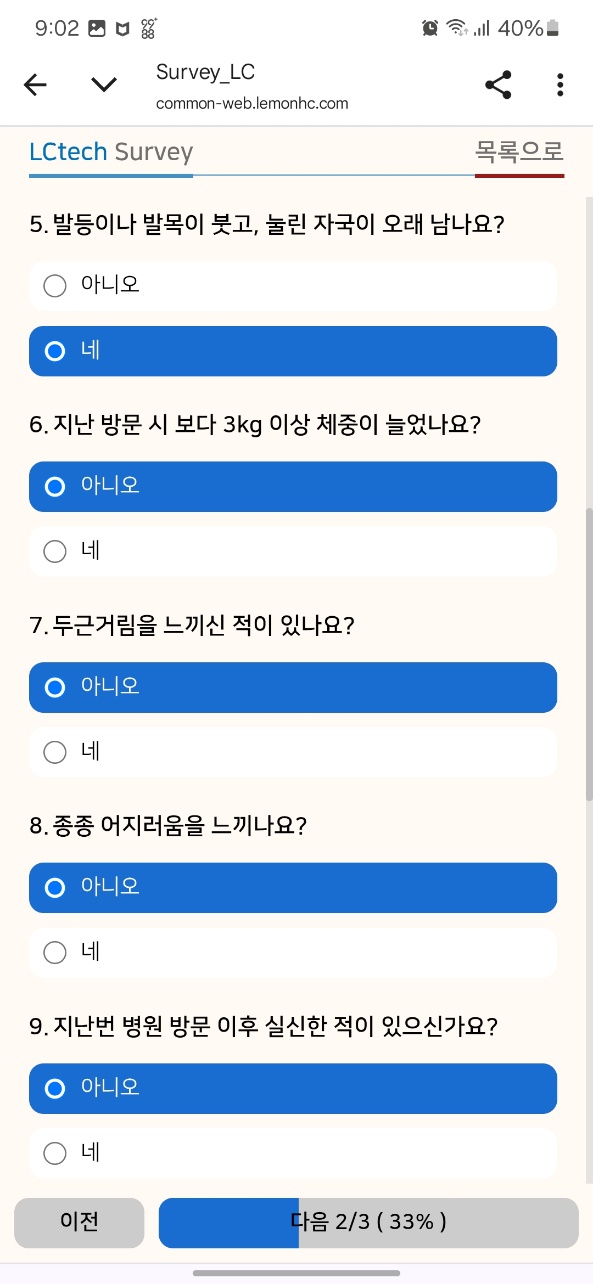


**Figure S1.** Previsit survey screen displaying patient-friendly NYHA class descriptions (left) and questions addressing heart failure symptoms (right).


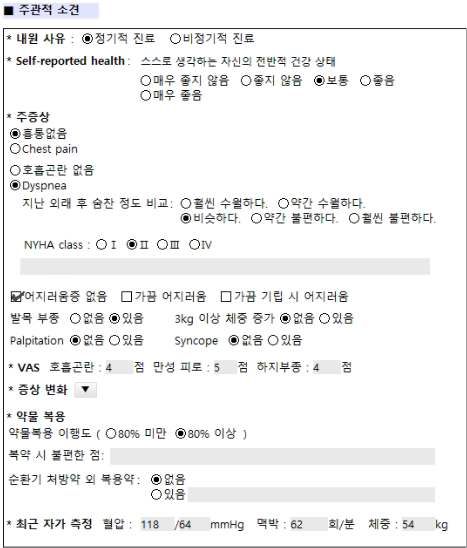


**Figure S2.** EMR^a^ screen with previsit survey responses integrated.

^a^EMR: Electronic medical record**Table S1.** Previsit questionnaire for new cardiovascular disease patients in English(Korean)

| No | Question | Response  rate | Yes |
| --- | --- | --- | --- |
| Q1-1 | Do you have any shortness of breath?  (숨찬 증상이 있나요?) | 138/138(100) | 51/138(37) |
| Q1-2 | How severe is your shortness of breath?  (얼마나 숨이 차나요?) | 48/51(94) |  |
|  | NYHA II(가벼운 운동시) |  | 16/48(33) |
|  | NYHA III(일상 생활 중) |  | 24/48(50) |
|  | NYHA IV(가만히 있어도) |  | 8/48(17) |
| Q3-1 | Do you have any chest pain?  (가슴 통증이 있나요?) | 138/138(100) | 45/138(32) |
| Q3-2 | How severe is your chest pain?  (얼마나 가슴이 아픈가요?) | 45/45(100) |  |
|  | I often feel it at rest, regardless of exercise or activity  (운동, 활동과는 무관하게 안정 상태에서도 종종 느낌) |  | 33/45(73) |
|  | During intense and sustained exercise  (강하고 빠른 운동을 해야 발생) |  | 3/45(7) |
|  | When climbing stairs or walking uphill quickly  (빠르게 계단을 오르거나 언덕을 오를 정도에서 발생) |  | 6/45(13) |
|  | It limits daily activities and occurs when climbing more than one flight of stairs  (일상 생활이 제한 되며, 계단 한 층 이상을 오르면 발생) |  | 2/45(4) |
|  | Physical activity is not possible without chest pain, and it persists even at rest  (흉통 없이 육체활동을 할 수 없으며, 휴식 시에도 지속) |  | 1/45(2) |
| Q4 | Do you have any palpitations?  (두근거리는 증상이 있나요?) | 137/138(99) | 48/137(35) |
| Q5 | Have you ever fainted?  (실신한 적이 있나요?) | 136/138(99) | 9/136(7) |
| Q6 | Have you ever been told that you need to manage your blood pressure?  (혈압관리가 필요하다는 얘기를 들은 적이 있나요?) | 136/138(99) | 55/136(40) |
| Q7 | Have you been diagnosed with any of the following diseases?  (아래의 질병을 진단 받은 적이 있나요?) | 138/138(100) | 83/138(60) |
| Q7-1 | Hypertension | 109/138(80) | 47/109(43) |
| Q7-2 | Diabetes Mellitus | 109/138(80) | 12/109(11) |
| Q7-3 | Dyslipidemia | 112/138(81) | 45/112(40) |
| Q7-4 | Coronary disease(Stable angina, Myocardial infarction) | 109/138(79) | 9/109(8) |
| Q7-5 | Arrhythmia | 107/138(78) | 11/107(10) |
| Q7-6 | Stroke | 108/138(78) | 6/108(6) |
| Q7-7 | Malignancy | 108/138(78) | 17/108(16) |
| Q8 | Do you consume alcohol?  (음주를 하나요?) | 137/138(99) |  |
|  | I quit drinking(끊었음) |  | 13/137(9) |
|  | Non-drinker(비음주자) |  | 70/137(51) |
|  | Yes(예) |  | 54/137(39) |
| Q9 | Do you smoke cigarettes?  (흡연을 하나요?) | 137/138(99) |  |
|  | Never smoked(비흡연자) |  | 80/137(58) |
|  | I quit smoking(끊었음) |  | 36/137(27) |
|  | Yes(현재 흡연중) |  | 21/137(15) |
| Q10 | Do you exercise regularly?  (평소에 규칙적인 운동을 하고 있나요?) | 136/138(99) | 58/136(43) |
| Q11 | Do you currently have a job?  (현재 직업이 있나요?) | 134/138(97) | 78/134(58) |
| Q12 | Do any of your parents, siblings, or children have high blood pressure, diabetes, high cholesterol, or coronary artery disease?  (부모, 형제, 자녀가 고혈압, 당뇨, 고지혈증, 관상동맥질환 중 하나라도 있나요?) | 138/138(100) | 87/138(63) |

**Table S2.** Previsit questionnaire for follow-up heart failure patients in English(Korean)

| No | Question | Response  Rate(%) | Yes(%) |
| --- | --- | --- | --- |
| Q1 | Was this visit a scheduled follow-up appointment  (이번 내원은 약속된 정기적 방문인가요?) | 148/151(98) | 144/148(97) |
| Q2 | How would you describe your overall health condition?  (자신의 전반적인 건강상태는 어떻다고 생각하세요?) | 151/151(100) |  |
|  | Very bad(매우 좋지 않음) |  | 10/151(7) |
|  | Bad(좋지 않음) |  | 47/151(31) |
|  | So so(보통) |  | 66/151(44) |
|  | Good(좋음) |  | 25/151(17) |
|  | Very good(매우 좋음) |  | 3/151(2) |
| Q3-1 | Do you have any shortness of breath?  (숨찬 증상이 있나요?) | 148/151(98) | 40/148(27) |
| Q3-2 | How severe is your shortness of breath?  (얼마나 숨이 차나요?) | 39/40(98) |  |
|  | NYHA II(가벼운 운동시) |  | 14/39(36) |
|  | NYHA III(일상 생활 중) |  | 18/39(46) |
|  | NYHA IV(가만히 있어도) |  | 7/39(18) |
| Q3-3 | Compared to your last visit, how has your shortness of breath changed?  (지난 번 병원 오셨을 때와 비교해서 숨찬 정도는 어떤가요?) | 40/40(100) |  |
|  | Breathing is much easier  (숨쉬기가 훨씬 수월하다) |  | 2/40(5) |
|  | Breathing is slightly easier  (숨쉬기가 약간 수월하다) |  | 4/40(10) |
|  | No change  (비슷하다) |  | 25/40(63) |
|  | Breathing is slightly more difficult  (숨쉬기가 약간 더 불편하다) |  | 9/40(23) |
|  | Breathing is much more difficult  (숨쉬기가 훨씬 불편하다) |  | 0/40(0) |
| Q4-1 | Do you have any chest pain?  (가슴 통증이 있나요?) | 145/151(96) | 14/145(10) |
| Q4-2 | How severe is your chest pain?  (얼마나 가슴이 아픈가요?) | 13/14(93) |  |
|  | I often feel it at rest, regardless of exercise or activity  (운동, 활동과는 무관하게 안정 상태에서도 종종 느낌) |  | 8/13(62) |
|  | During intense and sustained exercise  (강하고 빠른 운동을 해야 발생) |  | 2/13(15) |
|  | When climbing stairs or walking uphill quickly  (일상 생활이 제한 되며, 계단 한 층 이상을 오르면 발생) |  | 3/13(23) |
|  | It limits daily activities and occurs when climbing more than one flight of stairs  (흉통 없이 육체활동을 할 수 없으며, 휴식 시에도 지속) |  | 0/13(0) |
|  | Physical activity is not possible without chest pain, and it persists even at rest |  | 0/13(0) |
| Q5 | Do you have swelling in your feet or ankles that leaves a lasting indentation when pressed?  (발등이나 발목이 붓고, 눌린 자국이 오래 남나요?) | 144/151(95) | 22/144(15) |
| Q6 | Has your weight increased by more than 3 kg since your last visit?  (지난 방문 시보다 3kg이상 체중이 늘었나요?) | 144/151(95) | 16/144(11) |
| Q7 | Have you experienced heart palpitations?  (두근거림을 느끼신 적이 있나요?) | 140/151(93) | 35/140(25) |
| Q8 | Do you often feel dizzy?  (종종 어지러움을 느끼나요?) | 139/151(92) | 56/139(40) |
| Q9 | Have you fainted since your last hospital visit?  (지난 번 병원 방문 이후 실신한 적이 있으신가요?) | 138/151(91) | 3/138(2) |
| Q10 | Have you taken more than 80% of your prescribed medications?  (처방약은 80% 이상 챙겨 드셨습니까?) | 135/151(89) | 128/135(95) |
| Q11 | If you have any difficulties with medication intake, please describe briefly.  (약 복용에 불편한 점이 있으면 간략히 적어 주세요.) | 56/151(37) | 16/56(29) |
| Q12-1 | Are you taking any medications other than those prescribed by Korea University Guro hospital?  (저희 처방 외 다른 약도 드시는게 있나요?) | 135/151(89) | 72/135(53) |
| Q12-2 | If so, please list them to the best of your knowledge.  (드시는게 있다면, 아는 대로 적어주세요.) | 58/72(81) |  |
| Q13 | Please record your most recently measured blood pressure (systolic/diastolic mmHg).  가장 최근 측정한 혈압을 작성해주세요, 혈압 수축기/이완기 mmHg을 하단에 순서대로 작성해주세요. | 85/151(56) |  |
| Q14 | Please record your most recently measured pulse rate (beats per minute).  (가장 최근 측정한 맥박수를 작성해주세요(수/분)) | 76/151(50) |  |
| Q15 | Please record your most recently measured weight.  (가장 최근 측정한 체중을 작성해주세요.) | 116/151(77) |  |
| Q16 | If you have any additional information to share with the medical staff, please write freely.  (기타 의료진에게 전달하고 싶은 내용을 자유롭게 써주세요) | 42/151(28) |  |
| Q17 | Please rate the severity of the following symptoms on a scale of 0 to 10:  (호흡곤란, 만성피로, 하지부종 3가지 증상에 대한 점수를 작성해주세요.) |  |  |
|  | Shortness of breath (0-10)  호흡곤란 정도(0-10) | 135/151(89) |  |
|  | Chronic fatigue (0-10)  만성피로 정도(0-10) | 136/151(90) |  |
|  | Leg swelling (0-10)  하지부종 정도(0-10) | 133/151(88) |  |

**Table S3.** Postvisit satisfaction survey for patients in English(Korean), n=76

| No | Category | Question | Response  Rate(%) | Agree, Strongly agree/No. answered(%) |
| --- | --- | --- | --- | --- |
| Q1 | Appropriateness | Do you think KakaoTalk and text messages are appropriate notification methods?  (카카오톡과 문자가 알림 방식으로 적절하다고 생각하십니까?) | 76/76(100) | 70/76(92) |
| Q2 |  | Do you think the number of questions is appropriate?  (문항 수는 적절하다고 생각하십니까?) | 76/76(100) | 67/76(88) |
| Q3 | Acceptability | Was it easy to understand the questions?  (문항을 이해하기에 쉬웠습니까?) | 76/76(100) | 68/76(89) |
| Q4 |  | Were you able to complete the questions without assistance?  (타인의 도움 없이 문항작성을 완료할 수 있었나요?) | 76/76(100) | 66/76(87) |
| Q5 |  | Was the system easy to use?  (이용이 쉬웠습니까?) | 76/76(100) | 65/76(86) |
| Q6 | Satisfaction & Usefulness | Did using the service help with preparation before your outpatient visit?  (미리알리미를 이용하는게 외래 방문 전 준비에 도움이 됐나요?) | 76/76(100) | 66/76(87) |
| Q7 |  | Did the service help communicate your symptoms, condition, and health history to the medical staff?  (미리알리미가 의료진에게 자신의 증상, 상태, 건강 이력 등을 전달하는데 도움이 됐나요?) | 76/76(100) | 68/76(89) |
| Q8 |  | Do you think using the service helped the medical staff obtain more accurate information about you?  (미리알리미를 이용함으로써 의료진이 귀하에 대해 더 정확한 정보를 얻었다고 생각하십니까) | 76/76(100) | 64/76(84) |
| Q9 |  | Do you think the communication between you and the healthcare staff improved through the service?  (미리알리미를 통해 환자-의료진 간 소통이 개선됐다고 생각하십니까?) | 76/76(100) | 63/76(83) |
| Q10 |  | Do you think the service was helpful in your medical care?  (미리알리미가 귀하의 진료에 도움이 된다고 생각하십니까?) | 76/76(100) | 63/76(83) |
| Q11 |  | Are you satisfied with the overall use of the service for your medical visits?  (미리알리미를 활용한 진료 전반에 대해 만족하십니까) | 76/76(100) | 65/76(86) |
| Q12 |  | Would you be willing to continue using the service in the future?  (앞으로도 미리알리미를 이용할 의향이 있으십니까?) | 76/76(100) | 70/76(92) |
| Q13 |  | Would you recommend the service to friends or other patients?  (친지나 다른 환자들에게도 미리알리미 이용을 추천하시겠습니까?) | 76/76(100) | 64/76(84) |

**Table S4.** Postvisit satisfaction survey for nurses in English(Korean), n=8

|  | Category | Question | Yes, Strongly Yes/No. answered (%) | Median(25%, 75%) |
| --- | --- | --- | --- | --- |
| Q1 | Overall Satisfaction | Are you satisfied with the overall medical care provided using [MiriAlimi]?  [미리알리미]를 활용한 진료 전반에 대하여 만족하십니까? | 7/8(87.5) | 4(4,4) |
| Q2 | Appropriateness | Do you think the number and items in [MiriAlimi] are appropriate?  [미리알리미]의 문항수와 항목은 적절하다고 생각하십니까? | 7/8(87.5) | 4(4,4) |
| Q3 | Clinical Utility | \|  \| \| --- \| \| Did the patient self-report via [MiriAlimi] help in identifying the patient? \| \|   [미리알리미]를 통한 환자의 자가보고가 환자 파악에 도움이 됐습니까? | 7/8(87.5) | 4(4,4.25) |
| Q4 |  | Was it easier to manage patients using [MiriAlimi] compared to when it was not used?  외래 전 [미리알리미]를 활용한 환자의 응대가 그렇지 않은 경우보다 더 수월했나요? | 7/8(87.5) | 4(4,5) |
| Q5 | Improved workflow | Did [MiriAlimi] help reduce the time required for pre-visit preparation in outpatient care? [미리알리미]가 외래진료 사전준비시간 단축에 도움이 되었습니까? | 7/8(87.5) | 4.5(4,5) |
| Q6 |  | Did [MiriAlimi] contribute to reducing the time spent with patients?  [미리알리미]가 환자응대시간 단축에 도움이 되었습니까? | 7/8(87.5) | 4(4,5) |
| Q7 | Overall satsfaction | Do you agree with the continued use and expansion of [MiriAlimi]?  [미리알리미] 사용의 지속 및 확대에 동의하십니까? | 7/8(87.5) | 4(4,5) |
| Q8 |  | Free comments | Add self-measurement and recording of physical measurements and vital signs | |

**Table S5.** Postvisit satisfaction survey for cardiologists in English(Korean), n=2

|  | Category | Question | Yes, Strongly Yes/No. answered (%) |
| --- | --- | --- | --- |
| Q1 | Clinical Utility | Did the patient self-report through [MiriAlimi] help in identifying the patient?  [미리알리미]를 통한 환자의 자가보고가 환자 파악에 도움이 됐습니까? | 2/2(100) |
| Q2 |  | Did you incorporate the patient self-report from [MiriAlimi] into the medical record?  [미리알리미]를 통한 환자의 자가보고를 의무기록 작성에 반영했습니까? | 2/2(100) |
| Q3 |  | Did incorporating the patient self-report into the medical record help in increasing time for communication with the patient or for physical examinations?  [미리알리미]를 통한 환자의 자가보고 의무기록 반영이 환자와의 소통 시간 혹은 신체검사 등을 위한 시간을 늘리는데 도움이 되었나요? | 2/2(100) |
| Q4 | Perceived Patient Impact | Do you think patients find [MiriAlimi] useful?  환자들이 [미리알리미]를 유용하게 느낀다고 생각하시나요? | 2/2(100) |
| Q5 |  | Do you think patients visited the outpatient clinic better prepared when using [MiriAlimi]?  [미리알리미]를 활용하면서 환자들이 좀더 준비된 채 외래 방문했다고 생각하십니까? | 2/2(100) |
| Q6 |  | Do you think patients became more interested in their medical records when using [MiriAlimi]?  [미리알리미]를 활용하면서 환자들이 자신의 의무기록에 좀더 관심을 가지게 됐다고 생각하시나요? | 2/2(100) |
| Q7 |  | Did patients ask more health-related questions when using [MiriAlimi]?  [미리알리미]를 활용하면서 환자들이 자신의 건강 관련 질문을 더 많이 했나요? | 1/2(50) |
| Q8 |  | Do you think continuing to use [MiriAlimi] would benefit patients?  환자들이 [미리알리미]를 계속 활용하는 게 그 분들께 도움이 될까요? | 2/2(100) |
| Q9 |  | Do you think communication between patients and healthcare providers improved through [MiriAlimi]? [미리알리미]를 통해 환자-의료진 간 소통이 개선됐다고 생각하십니까? | 2/2(100) |
| Q10 | Clinical utility | Do you think [MiriAlimi] was helpful in your practice?  [미리알리미]가 귀하의 진료에 도움이 된다고 생각하십니까? | 2/2(100) |
| Q11 |  | Was the use of [MiriAlimi] efficient in medical practice?  [미리알리미]의 활용이 진료에 효율적이었습니까? | 2/2(100) |
| Q12 | Overall Satisfaction | Are you satisfied with the overall use of [MiriAlimi] in medical practice?  [미리알리미]를 활용한 진료 전반에 대해 만족하십니까? | 2/2(100) |
| Q13 |  | Would you recommend [MiriAlimi] to other physicians?  [미리알리미]를 다른 의사들에게도 추천하시겠습니까? | 2/2(100) |
| Q14 | Improved Workflow | Did the use of [MiriAlimi] shorten your consultation time?  [미리알리미]를 활용하면서 진료 시간이 감소했나요? | 1/2(50) |
| Q15 |  | Free comments | Not available |
